# Implementation Gaps in Public Outpatient Drug Programs: A Survey of Physicians in Urban Primary Care in Kazakhstan

**DOI:** 10.3390/ijerph23030279

**Published:** 2026-02-24

**Authors:** Kapiza Zhanzhigitova, Bibikhan Yeraliyeva, Zhanar Buribayeva, Natalya Cheboterenko, Nurken Abdiyev, Bibigul Kiyekova, Gulnara Erkinbekova, Guldana Nurgazieva

**Affiliations:** 1 Department of Public Health and Social Sciences, Kazakhstan Medical University “Higher School of Public Health”, Almaty 050060, Kazakhstan; dfarm22@mail.ru; 2 Department of Epidemiology, Evidence-Based Medicine and Biostatistics, Kazakhstan Medical University “Higher School of Public Health”, Almaty 050060, Kazakhstan; 3 Department of Clinical Pharmacology, S.D. Asfendiyarov Kazakh National Medical University, Almaty 050000, Kazakhstan; buribayeva.zhanar@kaznu.kz (Z.B.); kiekova.b@kaznmu.kz (B.K.); erkinbekova.g@kaznmu.kz (G.E.); nurgaziyeva.g@kaznmu.kz (G.N.); 4 Department of Nursing, S.D. Asfendiyarov Kazakh National Medical University, Almaty 050000, Kazakhstan; 5 Consortium for Scientific and Medical Research, Los Angeles, CA 90001, USA; corsumnews@gmail.com; 6 Private Hospital International Almaty, Almaty 050000, Kazakhstan; abdievnm@gmail.com

**Keywords:** outpatient drug provision, primary healthcare, physician awareness, health policy implementation, access to medicines, pharmaceutical governance

## Abstract

**Highlights:**

**Public health relevance—How does this work relate to a public health issue?**

- Outpatient drug provision is a core determinant of treatment continuity and equity in primary healthcare systems, particularly for patients with chronic conditions.
- This study examines how physician-level awareness and everyday clinical practices shape real-world access to publicly funded medicines in an urban health system.

**Public health significance—Why is this work of significance to public health?**

- The findings demonstrate that implementation failures in outpatient drug programs are primarily driven by systemic inefficiencies, suggesting that organizational and informational barriers take precedence over insufficient public financing. By focusing on physicians as frontline implementers, the study addresses a critical but underexplored dimension of pharmaceutical governance in upper-middle-income countries.

**Public health implications—What are the key implications or messages for practitioners, policy makers and/or researchers in public health?**

- Strengthening internal communication, administrative clarity, and pharmaceutical policy training for physicians may substantially improve the effectiveness of outpatient drug provision without increasing budgets.
- Physician-level implementation processes should be systematically incorporated into the design, monitoring, and evaluation of pharmaceutical policies in primary care.

**Abstract:**

Background: Outpatient drug provision is a critical component of primary healthcare systems and a key determinant of treatment continuity, adherence, and equity, yet the effectiveness of publicly funded outpatient drug programs often depends on how policies are implemented at the point of care. This study examined physician awareness, practical experience, and perceived barriers related to outpatient drug provision and drug cost compensation mechanisms in urban primary care settings in Kazakhstan. Methods: A descriptive cross-sectional survey was conducted between September and December 2024 among 380 physicians working in all 33 state-owned urban polyclinics in Almaty, using a structured author-developed questionnaire. Descriptive statistics and Pearson’s chi-square tests were applied to assess associations between physician characteristics and awareness levels. Results: Only 44.0% of physicians confirmed the existence of outpatient drug cost compensation mechanisms in their polyclinics, while 26.0% believed that no such mechanisms existed and 30.0% were unable to provide a definitive answer, indicating that 56.0% lacked accurate awareness. Limited medicine availability and recurrent shortages were frequently reported, with half of physicians advising patients to purchase medicines out of pocket. Physician awareness was significantly associated with professional experience and specialty (*p* < 0.001). Conclusions: These findings indicate a substantial physician-level implementation gap in outpatient drug provision, suggesting that organizational and informational barriers—rather than insufficient public financing—are the primary drivers, highlighting the need for strengthened governance.

## 1. Introduction

Access to essential medicines at the outpatient level is a key determinant of treatment continuity, clinical outcomes, and patient adherence, particularly within primary healthcare systems [1,2]. Globally, nearly two billion people lack access to essential medicines, particularly in low- and middle-income countries [2]. The 2030 Agenda for Sustainable Development identifies access to essential medicines as a prerequisite for achieving universal health coverage and reducing health-related inequalities [1]. Empirical evidence shows that free or subsidized provision of medicines reduces financial barriers and improves adherence to prescribed therapy, especially among socially vulnerable populations [2].

Primary healthcare systems increasingly bear responsibility for the long-term management of chronic non-communicable diseases requiring sustained outpatient pharmacotherapy [3]. Population aging and the growing prevalence of chronic conditions have increased demand for continuous access to medicines in ambulatory care, placing pressure on national outpatient drug provision systems [4]. Consequently, the organization and implementation of outpatient pharmaceutical supply have become important issues in public health policy and health system performance.

In Kazakhstan, outpatient drug provision operates within the framework of the Guaranteed Volume of Free Medical Care, whereby eligible patients receive medicines prescribed by primary care physicians [5]. National data indicate that public spending on free and reimbursed outpatient medicines reached approximately USD 435.5 million in 2022, covering 2.2 million patients within the Statutory Free Medical Assistance and Compulsory Social Health Insurance programs [6]. Despite this investment, patient satisfaction remains limited: only 22% of respondents reported full satisfaction with the organization of free medicine supply, while shortages, waiting times, and insufficient information were the most frequently reported concerns [7,8]. These findings suggest that financial coverage alone does not guarantee effective access to medicines in routine outpatient practice.

Comparable challenges are observed across Central Asia, where public financing represents a limited share of pharmaceutical expenditure and outpatient medicines are often financed through private out-of-pocket payments [9,10,11,12]. Access challenges also persist in high-income health systems, where shortages of essential outpatient medicines, particularly low-cost drugs, continue to occur despite established reimbursement mechanisms [13,14,15,16,17]. Together, these findings highlight that effective access to medicines depends not only on financing and policy design but also on implementation and governance processes.

Although system-level challenges in outpatient drug provision are well documented, most research focuses on patients, supply chains, or financing mechanisms. Far less attention has been paid to physicians as frontline actors responsible for implementing pharmaceutical policies in everyday clinical practice [18,19]. Primary care physicians issue prescriptions, inform patients about eligibility for publicly funded medicines, and make practical decisions when medicines are unavailable. Their awareness of drug cost compensation mechanisms and understanding of administrative procedures directly shape how pharmaceutical policies function at the point of care [20,21]. Domestic studies also point to persistent organizational and regulatory weaknesses in outpatient drug provision, including fragmented procurement processes and limited administrative transparency [18,19,20,21,22,23].

Kazakhstan’s predominantly public healthcare system, which evolved from the Semashko model and is currently undergoing reforms aimed at strengthening primary healthcare and advancing universal health coverage, relies heavily on primary care physicians for outpatient prescribing and implementation of pharmaceutical policies [24,25,26]. As of 2022, more than 6100 health facilities were operating nationwide, including approximately 5700 primary healthcare facilities responsible for outpatient prescribing and patient eligibility for publicly funded medicines [25]. Physician density remains substantially higher in urban than rural areas (57.1 vs. 17.2 per 10,000 population), underscoring the central role of urban primary care physicians in policy implementation [26].

To examine physician-level implementation processes, this study is situated within the field of implementation science and conceptually informed by the Consolidated Framework for Implementation Research (CFIR), which provides a structured approach to understanding how health policies are translated into routine clinical practice [27,28]. Understanding how outpatient drug policies are translated into routine clinical practice is therefore essential for improving access, continuity of care, and equity [29,30,31]. However, empirical evidence on physician awareness, decision-making, and perceived barriers in outpatient drug provision remains limited in Central Asian primary healthcare settings.

In this study, physician awareness is defined as self-reported knowledge of the existence and functioning of outpatient drug cost compensation mechanisms within a polyclinic. Limited physician-level evidence constrains the development of targeted managerial and governance interventions and hampers efforts to address implementation gaps in outpatient pharmaceutical policy.

Accordingly, the objectives of this study were: (1) to assess physician awareness of outpatient drug cost compensation mechanisms in urban primary care; (2) to describe physicians’ practical experience with outpatient drug provision, including responses to medicine shortages; (3) to identify professional and organizational factors associated with physician awareness in urban polyclinics of Almaty, Kazakhstan.

## 2. Materials and Methods

### 2.1. Study Design

This study employed a descriptive cross-sectional design aimed at assessing physician-level awareness, practices, and perceived barriers related to outpatient drug provision within the publicly funded primary healthcare system. The study was explicitly designed to explore implementation processes at the point of care, focusing on physicians as frontline actors responsible for translating pharmaceutical policy into routine outpatient practice. A cross-sectional design was selected as the most appropriate methodological approach to capture prevailing implementation patterns, professional behaviors, and organizational constraints within a defined time frame, without attempting to infer causality. The study was designed and reported in accordance with the Strengthening the Reporting of Observational Studies in Epidemiology (STROBE) guidelines [32] for cross-sectional studies; the completed STROBE checklist is provided as Supplementary Table S2.

### 2.2. Study Setting

The study was conducted in Almaty, Kazakhstan, the country’s largest city by population and its principal urban healthcare center. Almaty has a population of approximately 2.2 million people. Almaty was selected for several methodological and substantive reasons: it has the largest concentration of primary healthcare facilities in the country; it serves a heterogeneous urban population, including patients with diverse socioeconomic and clinical profiles; outpatient drug provision in Almaty operates under the same national regulatory framework as other regions, while being exposed to higher service demand and organizational complexity.

At the time of the study, 33 state-owned urban polyclinics were operating in Almaty. Although all 33 state-owned urban polyclinics operating in Almaty were included in the study, these facilities differ in size, staffing levels, and patient volume. The inclusion of all public polyclinics allowed comprehensive institutional coverage of the urban primary healthcare sector, while acknowledging heterogeneity in organizational capacity and service load across facilities.

### 2.3. Study Population

The target population consisted of physicians employed in state-owned urban polyclinics and actively involved in outpatient clinical care. Eligible participants included:

- general practitioners,
- pediatricians,
- therapists,
- and other outpatient specialists involved in prescribing decisions.

Physicians were eligible if they:

1. were employed at one of the participating polyclinics during the study period;
2. provided outpatient care involving prescription of medicines;
3. agreed to participate voluntarily and provided informed consent.

No exclusion criteria were applied based on age, sex, specialty, or length of professional experience in order to capture the full diversity of physician roles and implementation contexts within primary care.

### 2.4. Sample Size Determination

The sample size was determined based on both statistical considerations and organizational feasibility. Given the exploratory nature of the study and the absence of prior local estimates of physician awareness of outpatient drug cost compensation mechanisms, a conservative prevalence estimate of 50% was assumed to maximize sample size requirements. In the absence of prior national or regional studies quantifying physician awareness of outpatient drug cost compensation mechanisms in Kazakhstan, a conservative expected prevalence of 50% was assumed for sample size estimation. Use of this conservative assumption reduces the risk of underestimating the required sample size and is recommended in exploratory health systems and implementation research [33,34]. Using a 95% confidence level and a margin of error of 5%, the minimum required sample size was calculated to be approximately 374 physicians. In practice, 380 physicians were recruited, representing nearly complete coverage of the calculated sample size and ensuring adequate statistical power for descriptive and bivariate analyses. Importantly, the sample was drawn from all 33 state-owned urban polyclinics in Almaty, strengthening the representativeness of the findings for large urban primary healthcare settings within Kazakhstan.

A facility-based census approach was applied, including all state-owned urban polyclinics in Almaty (*n* = 33). Within each polyclinic, physicians were recruited using convenience sampling based on availability and eligibility during the data collection period. Although polyclinics varied in size, staffing, and patient volume, no stratification, proportional allocation, or predefined quotas were applied.

### 2.5. Conceptual Framework and International Guidance

The development of the survey instrument and the overall methodological approach were informed by internationally recognized frameworks and guidance documents issued by the World Health Organization (WHO).

Specifically, the study drew on:

- the WHO conceptual framework on access to essential medicines, which defines access as a function of availability, affordability, appropriate use, and health system organization [35];
- the WHO/Health Action International (HAI) methodology for assessing medicine availability, pricing, and access, widely used in international comparative studies [36];
- WHO guidance on health systems governance and pharmaceutical policy implementation, emphasizing the role of frontline healthcare providers in translating policy into practice [37].

These frameworks informed the selection of questionnaire domains, focusing on physician awareness, medicine availability, prescribing practices under conditions of shortage, and organizational mechanisms of drug cost compensation.

Although these WHO tools are primarily designed for system-level or facility-level assessments, they provide a robust conceptual basis for examining physician-level implementation processes, which remain underexplored in many settings.

#### Implementation Science Framework (CFIR)

This study was conceptually guided by the Consolidated Framework for Implementation Research, a widely used determinant framework in implementation science that supports systematic identification of factors influencing the translation of health policies into routine clinical practice. CFIR was applied in a pragmatic and descriptive manner to structure the examination of physician-level implementation processes related to outpatient drug provision, rather than to assess causal pathways or implementation effectiveness.

Consistent with the study objectives and available data, selected CFIR domains were operationalized, including characteristics of individuals, inner setting, and implementation process. These domains informed questionnaire domain selection and guided interpretation of findings related to physician awareness, organizational context, and practical responses to medicine unavailability. A detailed mapping of study variables to CFIR domains is provided in Supplementary Table S1.

### 2.6. Development, Linguistic Adaptation, and Validation of the Questionnaire

Due to the absence of previously validated instruments specifically designed to assess physician awareness and implementation of outpatient drug provision policies in Central Asian primary healthcare settings, the questionnaire was developed de novo.

Importantly, prior sociological or implementation-focused surveys addressing outpatient pharmaceutical policy at the physician level had not been conducted in the study setting. Data were collected using a structured, author-developed questionnaire comprising 15 items organized into three thematic blocks. The first block included four questions on general physician characteristics (sex, age group, specialty, and length of professional experience); the second block comprised seven questions assessing awareness and implementation of the outpatient drug provision program, including sources of patient information, frequency and reasons for prescription refusal, medicine availability and waiting time, treatment interruption due to shortages, physician responses to medicine unavailability, and patient complaint pathways; and the third block included four questions addressing patient behavior and policy perceptions, such as preferences for original versus generic medicines and attitudes toward digital notification systems and alternative reimbursement models. The questionnaire included a combination of single-choice, categorical, open-ended questions depending on the construct assessed. The full questionnaire with all items and response options is provided in Supplementary Table S3.

Given the bilingual working environment of Kazakhstan’s healthcare system, the questionnaire was developed simultaneously in Russian and Kazakh. A parallel bilingual development approach was employed rather than post hoc translation. This involved:

1. concurrent drafting of questionnaire items in both languages;
2. independent review of each language version by bilingual public health researchers and practicing physicians;
3. reconciliation of wording differences through consensus to ensure conceptual equivalence.

This process ensured linguistic clarity, minimized misinterpretation, and enhanced inclusiveness of physicians regardless of their primary working language.

The questionnaire underwent several validation steps appropriate for an exploratory, descriptive implementation study:

- Content validity was established through alignment with WHO frameworks, review of national regulatory documents governing outpatient drug provision, and expert consultation with public health specialists and clinicians.
- Face validity was assessed during expert review and pilot testing, ensuring that questions were clear, relevant, and appropriate for capturing physician awareness and practices.
- Pilot testing was conducted among 15 outpatient physicians who were not included in the final sample. Feedback focused on clarity, relevance, response options, and completion time.

Minor wording refinements were made following pilot testing. No items were added or removed. Formal psychometric testing (Cronbach’s alpha) was not performed, as the questionnaire did not aim to measure a single latent construct using scaled items. Instead, it consisted of discrete, policy-relevant questions addressing distinct aspects of outpatient drug provision, which is consistent with methodological standards for implementation and health systems research. Questionnaire domains were mapped to relevant CFIR domains to ensure conceptual alignment with implementation science principles (Supplementary Table S1). The questionnaire primarily focused on organizational and informational determinants of outpatient drug provision, including communication processes, administrative procedures, and medicine availability; workload-related factors such as time constraints or patient volume were not assessed as separate variables.

### 2.7. Data Collection Procedures

Data collection was conducted between September and December 2024 in participating urban polyclinics. All physicians who were invited to participate agreed to complete the questionnaire. Thus, the number of invited physicians was equal to the number of respondents (*n* = 380), resulting in a response rate of 100%.

Following institutional approval, physicians were approached on-site during routine working hours. The data collection process followed a standardized protocol:

1. Initial briefing: Eligible physicians received a brief verbal explanation of the study objectives, procedures, and voluntary nature of participation.
2. Informed consent: Physicians who agreed to participate were provided with a written informed consent form outlining confidentiality safeguards, the absence of personal identifiers, and the right to withdraw at any time without consequences.
3. Questionnaire administration: After providing written informed consent, participants were given a paper-based questionnaire in their preferred language (Russian or Kazakh). In facilities where electronic completion was feasible, a digital version of the questionnaire was provided.
4. Completion conditions: Questionnaires were completed independently, without the presence of supervisors or researchers, to reduce social desirability bias.
5. Time required: The average time required to complete the questionnaire was 11–13 min, as determined during pilot testing.
6. Collection and review: Completed questionnaires were collected immediately and reviewed for completeness. No follow-up or repeated contact with participants was required. No financial or non-financial incentives were offered for participation.

### 2.8. Outcome and Explanatory Variables

The primary outcome variable was physician awareness of outpatient drug cost compensation mechanisms. Awareness was assessed through a direct question asking whether such a mechanism existed within the respondent’s polyclinic.

Responses were categorized as: aware, not aware, unable to answer.

For analytical interpretation, lack of awareness and uncertainty were considered indicators of implementation failure. This interpretation is consistent with implementation research, where lack of awareness and uncertainty among frontline providers are considered indicators of implementation failure rather than individual-level deficits.

Explanatory variables included:

- length of professional experience (≤5 years, 6–10 years, >10 years);
- medical specialty (general practitioners, pediatricians, therapists, other specialties);
- sociodemographic characteristics (sex and age group).

Physician awareness was operationalized as a categorical variable based on self-reported knowledge of the existence of an outpatient drug cost compensation mechanism within the respondent’s polyclinic.

### 2.9. Data Management and Statistical Analysis

Data were entered and cleaned using Microsoft Excel (Microsoft Corporation, Redmond, WA, USA). Statistical analysis was performed using IBM SPSS Statistics for Windows, Version 26.0 (IBM Corp., Armonk, NY, USA).

Categorical variables were summarized using frequencies and percentages. Group comparisons were performed using Pearson’s chi-square test. Statistical significance was defined as *p* < 0.05. Multivariable analysis was not conducted due to the exploratory nature of the study and the absence of linked facility-level or policy-level indicators at the individual respondent level.

Given the absence of individual-level raw data linking all variables at the respondent level, further analysis relied on stratified and adjusted interpretation based on aggregated data, examining the consistency of associations across experience and specialty strata. The analytical focus was intentionally limited to physician-level variables, as facility-level system indicators such as polyclinic size, physician-to-population ratios, or financing data were not available at the individual respondent level and could not be reliably linked to survey responses.

### 2.10. Ethical Considerations

The study protocol was approved by the Ethics Committee of the Kazakhstan Medical University “Higher School of Public Health” (Protocol No. 2, dated 22 May 2024).

The study was conducted in accordance with the Declaration of Helsinki. Written informed consent was obtained from all participants. Confidentiality and anonymity were ensured throughout data collection, storage, and analysis.

## 3. Results

### 3.1. Characteristics of the Study Population

A total of 380 physicians from 33 urban polyclinics in Almaty participated in the study (Table 1).

The study sample predominantly consisted of female physicians, reflecting the gender structure of the urban primary healthcare workforce. Most respondents were clinically active, working-age physicians, with nearly 70% aged under 40 years. More than half of participants were early-career physicians (≤5 years of experience), highlighting the relevance of training and information dissemination mechanisms. General practitioners constituted half of the sample, underscoring the central role of primary care physicians in outpatient drug provision and policy implementation. The inclusion of physicians from all 33 state-owned urban polyclinics ensured broad institutional coverage of the public primary healthcare sector in Almaty. The predominance of early-career physicians reflects the current workforce structure in urban primary care and is particularly relevant for assessing the effectiveness of information dissemination and onboarding mechanisms related to outpatient drug policies. The balanced representation of general practitioners, pediatricians, and therapists allows meaningful comparisons across professional roles directly involved in outpatient prescribing and policy implementation.

### 3.2. Sources of Information and Prescribing Practices

**Figure 1:**
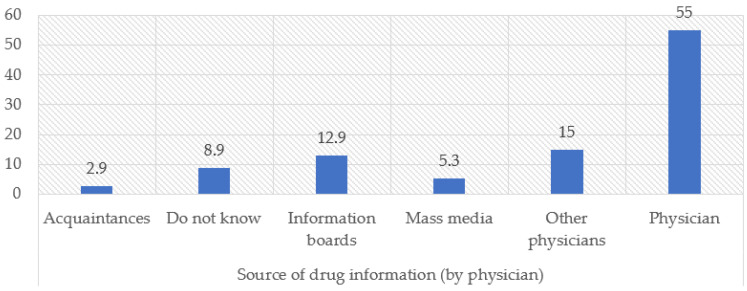
Where do patients obtain information about free medications, according to doctors?

According to physicians’ reports, the treating physician was identified as the primary source of information on free medicines for patients in more than half of cases (55.0%, *n* = 209). Other physicians were cited as an information source by 15.0% of respondents (*n* = 57), while informational materials such as posters or stands were mentioned by 12.9% (*n* = 49). Mass media played a limited role (5.3%, *n* = 20). Notably, 9.0% of physicians indicated that patients were not informed about free medicine options at all (Figure 1).

**Figure 2:**
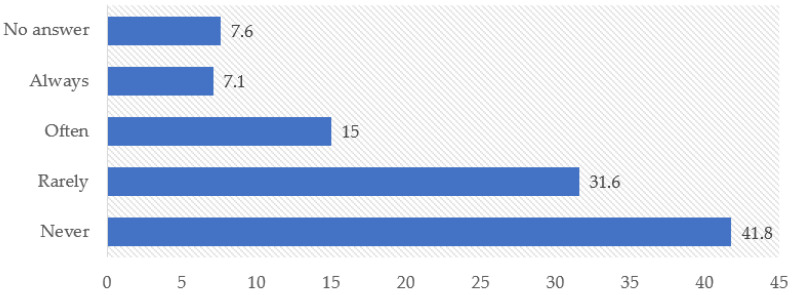
Refusal to issue a prescription to a patient for medication.

**Figure 3:**
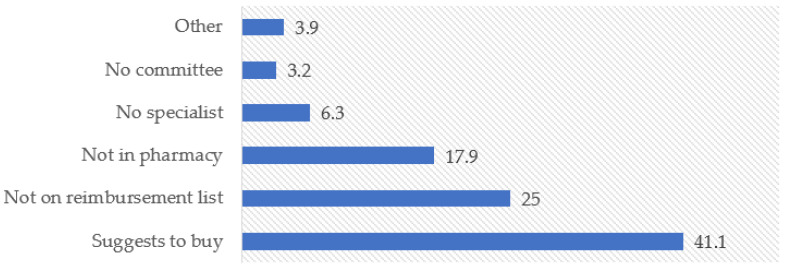
Reasons for refusal to issue prescriptions.

Most physicians reported that they either never refused to issue prescriptions (41.8%, *n* = 159) or did so rarely (31.6%, *n* = 120). However, refusals occurred frequently or on a regular basis among a smaller but non-negligible proportion of respondents (22.1% combined). The most common reason for refusing to issue a prescription was advising patients to purchase the medicine at their own expense (41.1%, *n* = 156), followed by the absence of the medicine from the free provision list (25.0%, *n* = 95) and physical unavailability in pharmacies (17.9%, *n* = 68) (Figure 2 and Figure 3).

The findings indicate that physicians serve as the primary intermediaries between outpatient drug policy and patients. With more than half of respondents identifying the treating physician as the main source of information on free medicines, access to policy-relevant information for patients largely depends on physician awareness and communication practices. The limited role of institutional information channels, such as informational materials and mass media, further underscores the central role of physicians in mediating access to outpatient pharmaceutical benefits.

### 3.3. Medicine Availability and Patient Behavior

**Figure 4:**
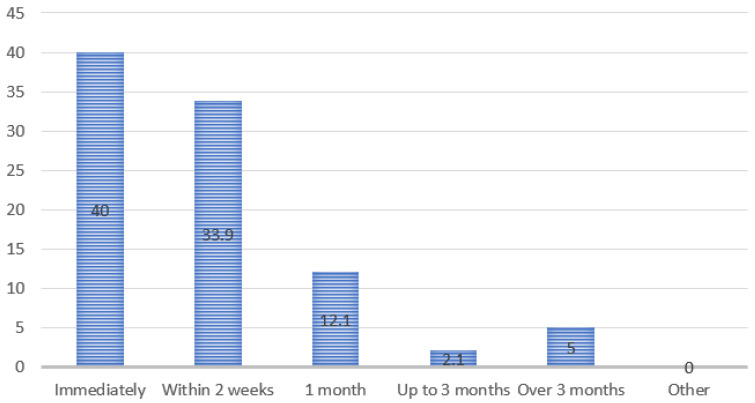
Waiting time for medicines.

Immediate access to prescribed medicines was reported by 40.0% of physicians (*n* = 152). Waiting periods of up to two weeks were reported by 34.0% (*n* = 129), while delays of one month were noted by 12.0% (*n* = 46). Longer delays of up to three months or more were reported in 7.0% of cases (*n* = 27), indicating episodic but clinically relevant disruptions in medicine availability (Figure 4).

Physicians reported that treatment interruption due to medicine unavailability occurred rarely (36.1%, *n* = 137) or occasionally (23.9%, *n* = 91). At the same time, 12.9% of respondents (*n* = 49) indicated that such interruptions occurred regularly, raising concerns regarding continuity of care.

In situations of medicine shortage, physicians most commonly recommended that patients purchase the medicine independently (50.0%, *n* = 190). Less frequently, physicians advised patients to wait for availability (35.0%, *n* = 133) or offered a therapeutic alternative (10.0%, *n* = 38). These patterns suggest a lack of standardized clinical algorithms for managing medicine shortages at the outpatient level.

Delays exceeding one month, although reported by a minority of physicians, are clinically significant in the context of chronic disease management and indicate vulnerabilities in continuity of outpatient pharmacotherapy.

### 3.4. Patient Preferences Regarding Medicines

Nearly half of physicians reported that patients always requested original (brand-name) medicines (45.8%, *n* = 174), while an additional 26.0% (*n* = 99) indicated that such requests occurred frequently. In contrast, only 3.9% of respondents (*n* = 15) stated that patients never requested original medicines.

Attitudes toward generic medicines were more heterogeneous. While 38.0% of physicians reported that patients rarely refused generics (*n* = 144) and 22.1% reported no refusals (*n* = 84), a substantial proportion encountered frequent or consistent refusals (25.0%, *n* = 95). Additionally, 9.0% of physicians were unable to provide a clear assessment, suggesting variability in patient–physician communication regarding generic substitution (Table 2).

High patient demand for original medicines, combined with mixed acceptance of generics, adds an additional layer of complexity to outpatient prescribing under conditions of limited availability and may further constrain physicians’ ability to adhere to formulary-based prescribing policies.

### 3.5. Awareness of Outpatient Drug Cost Compensation Mechanisms

Among respondents, 44.0% (*n* = 167) confirmed the existence of a drug cost compensation mechanism within their polyclinic. In contrast, 26.0% (*n* = 99) believed that no such mechanism was in place. Most notably, 30.0% of physicians (*n* = 114) were unable to state whether a compensation mechanism existed in their institution. Taken together, these findings indicate that 56.0% of physicians either lacked awareness of the compensation mechanism or perceived it as absent. Given that outpatient drug cost compensation is formally regulated and publicly financed, this pattern reflects a substantial implementation gap related to communication and execution at the frontline level rather than the absence of formal policy.

### 3.6. Associations Between Physician Characteristics and Awareness

Statistically significant associations were observed between physician awareness of the compensation mechanism and both length of professional experience and specialty. Associations were assessed using Pearson’s chi-square test. Physicians with more than ten years of experience were significantly more likely to report awareness of the mechanism compared with those with less than five years of experience (χ^2^ = 28.95; df = 4; *p* < 0.001). Early-career physicians demonstrated the highest level of uncertainty, with one-third reporting that they did not know whether the mechanism existed (Table 3).

A similar pattern was observed across specialties (χ^2^ = 24.21; df = 6; *p* < 0.001). General practitioners were more likely to confirm awareness of the compensation mechanism, whereas therapists and physicians from other specialties more frequently reported uncertainty. These findings point to uneven dissemination of administrative information within primary healthcare institutions and highlight the role of professional position and experience in policy implementation.

Taken together, the findings summarized in Table 3 demonstrate that outpatient drug provision in urban primary care is constrained primarily by physician-level implementation barriers rather than by insufficient public financing. Limited awareness of compensation mechanisms, combined with inconsistent medicine availability and the absence of standardized responses to drug shortages, results in frequent reliance on patient out-of-pocket purchases. The observed associations between physician awareness, professional experience, and specialty further suggest that organizational communication within primary healthcare institutions remains fragmented, contributing to uneven policy implementation at the point of care.

An integrated examination of the results reveals a consistent pattern of physician-level implementation barriers in outpatient drug provision. Limited awareness of compensation mechanisms coexists with frequent medicine shortages and the absence of standardized clinical responses to unavailability. In this context, physicians frequently rely on out-of-pocket solutions for patients, effectively shifting the burden of access from the health system to individuals.

The convergence of information gaps, organizational constraints, and patient preferences suggests that outpatient drug provision functions as a discretionary rather than standardized process. These patterns are indicative of systemic implementation challenges at the primary care level rather than isolated operational failures.

## 4. Discussion

This study contributes to the literature on access to medicines and pharmaceutical governance by shifting the analytical focus from policy design and financing to physician-level implementation processes in urban primary care. Unlike prior studies that primarily examine patient-reported barriers, supply chains, or macro-level reimbursement frameworks, this research provides empirical evidence on how frontline physicians perceive, interpret, and operationalize outpatient drug provision policies in routine clinical practice. By conceptualizing physician awareness as a core governance variable, the study demonstrates that implementation gaps arise not from the absence of formal compensation mechanisms, but from weaknesses in internal communication, organizational learning, and procedural clarity at the point of care.

Using a comprehensive city-wide sample of public polyclinics in an upper-middle-income setting, this study offers novel, policy-relevant insights into how administrative and informational barriers shape real-world access to medicines, thereby extending existing frameworks on access to essential medicines and health system governance. The results indicate that access to outpatient medicines in everyday practice is shaped less by formal policy design than by how pharmaceutical rules are communicated, interpreted, and applied at the level of routine clinical work. Similar implementation patterns have been described in health systems research from low- and middle-income countries (LMICs), where formal pharmaceutical policies co-exist with heterogeneous operational practices at the point of care [38,39,40].

### 4.1. Physician Awareness and Access to Outpatient Medicines

One of the central findings of the study is the limited awareness of outpatient drug cost compensation mechanisms among physicians. Fewer than half of respondents (44.0%) reported being aware of such mechanisms, while the remaining physicians either believed that no mechanism existed (26.0%) or were unable to determine its presence (30.0%). Taken together, these findings suggest that a substantial proportion of frontline prescribers operate with incomplete information regarding available reimbursement arrangements.

This degree of uncertainty is consistent with evidence from low- and middle-income health systems, where weaknesses in provider-level knowledge, internal information flows, and governance mechanisms have repeatedly been identified as determinants of effective access to essential medicines. Recent systematic reviews of access to medicines in low-resource settings highlight persistent gaps in availability, affordability, and communication across health system levels, with structural bottlenecks in information dissemination and decision pathways contributing to inequitable access [38,40]. In contexts where outpatient pharmaceutical policies rely on frontline clinicians to operationalize eligibility rules and administrative procedures, deficiencies in physician awareness regarding policy details and organizational processes can translate directly into variation in medicine access between facilities and patient groups. This underscores the need to strengthen internal governance arrangements that support capacity building, information systems, and accountability at the provider level to ensure that formal benefit schemes achieve their intended outcomes.

### 4.2. Professional Experience and Organizational Learning

The association between physician awareness and professional experience observed in this study points to the importance of organizational learning processes within primary healthcare institutions. Physicians with longer clinical experience were more likely to report awareness of compensation mechanisms, suggesting that practical knowledge of administrative procedures may be acquired gradually through repeated interaction with institutional structures rather than through standardized training.

Comparable patterns have been reported in LMIC studies, which show that provider behavior and policy knowledge are often shaped by informal practices, peer exchange, and accumulated institutional experience rather than by formal policy documents alone [41,42]. Findings from WHO/HAI surveys further indicate that the effectiveness of pharmaceutical policy implementation frequently depends on locally embedded routines and communication practices [43]. In this context, variation in awareness reflects organizational dynamics rather than individual shortcomings.

### 4.3. Medicine Availability and Adaptive Clinical Practices

Medicine availability emerged as an important operational constraint. Only 40.0% of physicians reported immediate access to prescribed medicines, while approximately one-fifth described delays of one month or longer. Such delays are clinically relevant, particularly for patients with chronic conditions requiring continuous pharmacotherapy, and are widely documented in outpatient settings across LMICs [44,45].

When medicines were unavailable, half of the physicians reported advising patients to purchase medicines independently. This practice appears to represent an adaptive response aimed at avoiding treatment interruption rather than a departure from policy intent. However, reliance on out-of-pocket purchasing remains a well-established source of financial burden and inequity in LMIC contexts, even where public drug provision schemes exist [46,47,48].

Patient demand for original medicines and variable acceptance of generics further shaped outpatient prescribing practices. Similar patterns have been reported internationally, where trust in medicine quality, previous treatment experience, and perceptions of efficacy influence patient preferences and may complicate generic substitution policies [49,50,51]. These dynamics interact with supply constraints to affect real-world access outcomes.

### 4.4. Policy Frameworks and Everyday Practice

Despite substantial public investment in outpatient pharmaceutical provision, the findings suggest that financing and formal policy design alone are insufficient to ensure consistent access at the point of care. International evidence indicates that effective access to medicines depends on a combination of adequate financing, reliable supply, rational use, and functional governance mechanisms [40,52]. The World Health Organization has repeatedly highlighted that weaknesses in implementation and delivery systems can undermine pharmaceutical policy objectives, even in settings with well-defined regulatory frameworks [51].

Where pharmaceutical policies are insufficiently integrated into routine clinical and administrative workflows, frontline providers may rely on discretionary practices to manage uncertainty, resulting in variable access across patient groups [38,40].

### 4.5. Broader Relevance for LMIC and Upper-Middle-Income Health Systems

Although this study was conducted in a single metropolitan area, its findings are relevant to a wider group of LMIC and upper-middle-income countries with centrally regulated outpatient drug programs implemented through primary care. International assessments, including the Access to Medicine Index, suggest that disparities in medicine availability and affordability are often driven by system-level and organizational barriers rather than by the absence of formal policy commitments [52].

By focusing on physician-level implementation processes, this study adds to the growing body of evidence showing that access to medicines is shaped by everyday practices within healthcare organizations. Strengthening internal communication, embedding pharmaceutical policy into clinical training, and developing clearer procedural guidance for managing medicine shortages may help narrow the gap between formal entitlement and practical access [38,40,51].

### 4.6. Limitations

Several limitations of this study should be considered.

First, the cross-sectional design does not allow for causal inference. The observed associations between physician characteristics and awareness of outpatient drug cost compensation mechanisms should therefore be interpreted as indicative of implementation patterns rather than causal relationships. In addition, workload-related factors such as time constraints and patient volume were not included in the analysis and should be examined in future studies exploring physician-level implementation barriers in outpatient drug provision.

Second, although all state-owned urban polyclinics in Almaty were included, physician recruitment within facilities relied on convenience sampling. This may have introduced selection bias, as physicians who were present and available during data collection may differ systematically from those who did not participate. Nevertheless, the large sample size, inclusion of all public polyclinics, and representation of multiple specialties mitigate this risk and strengthen the internal validity of the findings for large urban primary care settings.

Third, physician awareness was assessed using self-reported responses, which may not fully capture actual knowledge or procedural competence. While anonymous data collection and the absence of supervisory oversight were intended to reduce social desirability bias, some degree of misclassification cannot be excluded. Importantly, however, perceived awareness is itself a meaningful indicator of implementation performance, as clinical decision-making is guided by what physicians believe to be operationally available.

Fourth, the study relied primarily on descriptive and bivariate analyses. Multivariable modeling was not performed due to the exploratory nature of the study and limitations in linking individual-level variables across all domains. Future research incorporating multilevel or longitudinal designs could further clarify the relative contribution of individual, organizational, and system-level factors to implementation outcomes.

Finally, the study focused on physician-level processes and did not directly assess patient outcomes or administrative performance indicators. This focus was intentional, given the study’s aim to examine frontline implementation dynamics. Integrating physician-, patient-, and system-level data represents an important direction for future research. Although CFIR was used to guide interpretation, not all CFIR domains and constructs were systematically measured. The framework was applied pragmatically to structure physician-level implementation findings rather than to conduct comprehensive theory testing, which should be addressed in future implementation-focused studies.

## 5. Conclusions

This study demonstrates that challenges in outpatient drug provision within urban primary healthcare are primarily rooted in implementation processes at the physician and organizational levels, rather than in the absence of formal policy frameworks or insufficient public financing.

A substantial proportion of physicians lacked accurate awareness of existing outpatient drug cost compensation mechanisms, highlighting weaknesses in internal communication and governance. In the absence of standardized organizational guidance, physicians frequently relied on pragmatic, discretionary strategies—most notably, recommending out-of-pocket purchasing—to maintain treatment continuity, with potential implications for equity and financial protection.

The findings suggest that improving outpatient drug provision does not necessarily require expansion of pharmaceutical budgets, but rather targeted interventions aimed at strengthening implementation. Such interventions include systematic dissemination of policy-relevant information, integration of pharmaceutical policy and administrative procedures into continuing medical education, development of standardized responses to medicine shortages, and clearer institutional accountability for policy execution at the primary care level.

Although grounded in the context of Kazakhstan, the results are relevant to other low- and middle-income and upper-middle-income health systems where outpatient drug programs are centrally regulated but implemented through frontline providers. By highlighting physician-level implementation dynamics, this study contributes to international debates on access to medicines and underscores the importance of governance and institutionalization in achieving equitable and effective outpatient pharmaceutical care.

## Figures and Tables

**Table 1:** Sociodemographic and professional characteristics of the study participants (*n* = 380).

Characteristic	Category	*n*	%
Sex	Female	274	72.1
	Male	106	27.9
Age (years)	<30	95	25.0
	30–39	114	30.0
	40–49	145	38.2
	50–59	15	3.9
	≥60	11	2.9
Professional experience (years)	≤5	209	55.0
	6–10	133	35.0
	>10	38	10.0
Specialty	General practitioners	190	50.0
	Pediatricians	95	25.0
	Therapists	76	20.0
	Other specialties *	19	5.0

* Other specialties include endocrinologists, gynecologists, and other outpatient specialists.

**Table 2:** Behavioral and organizational aspects related to drug provision (*n* = 380).

Variable	Category	Quantity (*n*)	%
Termination of treatment	Rarely	137	36.1%
	Sometimes	91	23.9%
	Never	57	15.0%
	Almost always	19	5.0%
	Always	49	12.9%
	Other	27	7.1%
Actions of a physician in the absence of drugs	Recommends buying	190	50.0%
	Advises to wait	133	35.0%
	Offers a replacement	38	10.0%
	Other	19	5.0%
Where do patients complain most often?	Help Desk	137	36.0%
	Head physician	103	27.0%
	UZ	65	17.0%
	Polyclinic	30	8.0%
	Website of the President	15	4.0%
	I’m having trouble	30	8.0%
Patients are asking for original drugs	Always	174	45.8%
	Often	99	26.0%
	Sometimes	68	17.9%
	Never	15	3.9%
	I’m having trouble	23	6.1%
Attitude to generics (according to the doctor)	Rarely refuse	144	38.0%
	Never refuse	84	22.1%
	Sometimes	72	18.9%
	Often	57	15.0%
	They don’t want it at all	38	10.0%
	I find it difficult to answer	34	9.0%

**Table 3:** Summary of key findings on outpatient drug provision in urban primary care.

Domain	Key Finding	Quantitative Result	Interpretation
Physician awareness	Limited awareness of drug cost compensation mechanisms	44.0% aware; 26.0% believe mechanism does not exist; 30.0% do not know	More than half of physicians (56.0%) are unable to correctly identify the existence of compensation mechanisms, indicating a major implementation gap
Policy implementation	Functional failure at the physician level	56.0% combined lack of awareness or misperception	The barrier lies not in the absence of policy, but in ineffective dissemination and implementation
Medicine availability	Delays and shortages are common	Only 40.0% report immediate access; up to 19.9% report delays ≥1 month	Medicine availability is inconsistent and may compromise treatment continuity
Physician response to shortages	Reliance on out-of-pocket solutions	50.0% recommend patients purchase medicines independently	Absence of standardized clinical algorithms for managing shortages
Patient behavior	Preference for original medicines	71.8% request originals always or frequently	Patient preferences further complicate prescribing under limited availability
Generics acceptance	Mixed attitudes toward generics	25.0% frequent refusals; 9.0% uncertain	Indicates communication gaps and variability in physician–patient interactions
Determinants of awareness	Experience and specialty matter	χ^2^ = 28.95 (experience); χ^2^ = 24.21 (specialty), *p* < 0.001	Administrative information is unevenly distributed within primary care

## Data Availability

The data presented in this study are available on request from the corresponding author. The data are not publicly available due to ethical and privacy considerations, as they contain sensitive information related to healthcare professionals and institutional practices.

## Supplementary Materials

The following supporting information can be downloaded at: https://www.mdpi.com/article/10.3390/ijerph23030279/s1, Table S1: Mapping of study variables to CFIR domains; Table S2: STROBE Checklist for Cross-Sectional Studies; Table S3: STROBE Checklist for Cross-Sectional Studies.

## References

[1] Nazar R., Meo M.S., Ali S. (2022). Role of public health and trade for achieving sustainable development goals. J. Public Aff..

[2] Kvarnström K., Westerholm A., Airaksinen M., Liira H. (2021). Factors Contributing to Medication Adherence in Patients with a Chronic Condition: A Scoping Review of Qualitative Research. Pharmaceutics.

[3] Östbring M.J., Eriksson T., Petersson G., Hellström L. (2021). Effects of a pharmaceutical care intervention on clinical outcomes and patient adherence in coronary heart disease: The MIMeRiC randomized controlled trial. BMC Cardiovasc. Disord..

[4] Schwarz T., Schmidt A.E., Bobek J., Ladurner J. (2022). Barriers to accessing health care for people with chronic conditions: A qualitative interview study. BMC Health Serv. Res..

[5] Zhussupova G., Aiypkhanova A., Zhaldybayeva S., Satmbekova D., Akhayeva T., Kaliyeva S. (2025). Evaluation of a national framework for rational use of medicines in Kazakhstan and its role in improving medicine use practices at the organizational and national levels. BMC Health Serv. Res..

[6] Nazarbayev A., Nurbakyt A., Omirbayeva B., Akhmetzhan A., Kosherbayeva L. (2024). Characteristics of High-Cost Beneficiaries of Prescription Drugs in Kazakhstan: A Cross-Sectional Study of Outpatient Data from 2022. Clin. Outcomes Res..

[7] Zhussupova G., Koikov V. (2024). An Analysis of the Adult Population’s Opinion in the Republic of Kazakhstan on Satisfaction with the Free Medicine Supply System. Astana Med. J..

[8] Shaki D., Aimbetova G., Baysugurova V., Kanushina M., Chegebayeva A., Arailym M., Merkibekov E., Karibayeva I. (2025). Level of Patient Satisfaction with Quality of Primary Healthcare in Almaty During COVID-19 Pandemic. Int. J. Environ. Res. Public Health.

[9] Moldoisaeva S., Kaliev M., Sydykova A., Muratalieva E., Ismailov M., Madureira Lima J., Rechel B. (2022). Kyrgyzstan: Health System Review. Health Syst. Transit..

[10] Rechel B., Sydykova A., Moldoisaeva S., Sodiqova D., Spatayev Y., Ahmedov M., Robinson S., Sagan A. (2023). Primary care reforms in Central Asia—On the path to universal health coverage?. Health Policy Open.

[11] Cho M.J., Haverkort E. (2023). Out-of-pocket health care expenditures in Uzbekistan: Progress and reform priorities. Rural Health-Investment, Research and Implications.

[12] Shaltynov A., Jamedinova U., Semenova Y., Abenova M., Myssayev A. (2024). Inequalities in Out-of-Pocket Health Expenditure Measured Using Financing Incidence Analysis (FIA): A Systematic Review. Healthcare.

[13] Hernandez I., Sullivan S.D., Hansen R.N., Fendrick A.M. (2024). Cheaper is not always better: Drug shortages in the United States and a value-based solution to alleviate them. J. Manag. Care Spec. Pharm..

[14] Kardas P., Bago M., Barnestein-Fonseca P., Garuolienė K., Granas A.G., Gregório J., Hadžiabdić M.O., Kostalova B., Leiva-Fernández F., Lewek P. (2022). Reimbursed medication adherence enhancing interventions in 12 european countries: Current state of the art and future challenges. Front. Pharmacol..

[15] Chakravarty S. (2020). Did the Medicare Prescription Drug Program Lead to New Racial and Ethnic Disparities? Examining Long-term Changes in Prescription Drug Access among Minority Populations. Soc. Work Public Health.

[16] Adetunji O., Oliver J.F., Parasrampuria S., Singson G., Beleche T. (2024). The Potential Role of the Nonprofit Pharmaceutical Industry in Addressing Shortages and Increasing Access to Essential Medicines and Low-Cost Medicines.

[17] Religioni U., Barrios-Rodríguez R., Requena P., Borowska M., Ostrowski J. (2025). Enhancing Therapy Adherence: Impact on Clinical Outcomes, Healthcare Costs, and Patient Quality of Life. Medicina.

[18] Wong W.B., Seetasith A., Hung A., Zullig L.L. (2023). Impact of list price changes on out-of-pocket costs and adherence in four high-rebate specialty drugs. PLoS ONE.

[19] Alavi A.S.R., Kazemnejad L.E., Sheikhtaheri A. (2022). Physicians’, and Pharmacists’, viewpoint on ambulatory electronic prescription system. J. Health Adm..

[20] Lobuteva A., Lobuteva L., Zakharova O., Gribova Y., Nesterova N., Avertseva I., Karpova M. (2024). Prospects for the development of the electronic prescription system in the conditions of the modern pharmaceutical market of Russia. BMC Health Serv. Res..

[21] Alsahali S., Almutairi G., Aedh R., Alanezi S., Almutairi H., Anaam M., Alshammari M., Alhabib A., Alowayed A., Abdulsalim S. (2023). Perceptions of Community Pharmacists toward the National E-Prescribing Service (Wasfaty) and Exploring the Benefits and Challenges of the Service: A Descriptive Study from Qassim Region, Saudi Arabia. Pharmacy.

[22] Samadbeik M., Ahmadi M., Sadoughi F., Garavand A. (2019). Main Elements of National Model of Electronic Prescription System from Physicians’ Point of View: A Case Study in a Developing Country. Iran. J. Pharm. Res..

[23] Hailiye Teferi G., Wonde T.E., Tadele M.M., Assaye B.T., Hordofa Z.R., Ahmed M.H., Hailegebrael S. (2022). Perception of physicians towards electronic prescription system and associated factors at resource limited setting 2021: Cross sectional study. PLoS ONE.

[24] Semenova Y., Lim L., Salpynov Z., Gaipov A., Jakovljevic M. (2024). Historical evolution of healthcare systems of post-soviet Russia, Belarus, Kazakhstan, Kyrgyzstan, Tajikistan, Turkmenistan, Uzbekistan, Armenia, and Azerbaijan: A scoping review. Heliyon.

[25] Jobalayeva B., Glushkova N., Khismetova Z., Tanatarova G., Zhagiparova Z., Semenova Y. (2025). Past, current status, and future trends of the rural healthcare network in the Republic of Kazakhstan. Sci. Rep..

[26] Shaltynov A., Abenova M., Baibussinova A., Semenova Y., Omarov N., Tanatarova G., Sepbossynova A., Rocha J. (2025). Inequality in the Distribution and Utilization of Healthcare Resources in Kazakhstan (2002–2023): A Spatiotemporal Analysis. Int. J. Environ. Res. Public Health.

[27] Damschroder L.J., Aron D.C., Keith R.E., Kirsh S.R., Alexander J.A., Lowery J.C. (2009). Fostering implementation of health services research findings into practice: A consolidated framework for advancing implementation science. Implement. Sci..

[28] Damschroder L.J., Reardon C.M., Widerquist M.A.O., Lowery J. (2022). The updated Consolidated Framework for Implementation Research based on user feedback. Implement. Sci..

[29] Traylor D.O., Anderson E.E., Etsey M., Fenton B., Cheema N., McCampbell D., Patel D., Clark B. (2025). Practical Care Coordination for Primary Care Providers: Bridging the Gap Between Clinical Practice and Patient Outcomes.

[30] Endalamaw A., Khatri R.B., Mengistu T.S., Erku D., Wolka E., Zewdie A., Assefa Y. (2024). A scoping review of continuous quality improvement in healthcare system: Conceptualization, models and tools, barriers and facilitators, and impact. BMC Health Serv. Res..

[31] El-Harakeh A., Haley S.J. (2022). Improving the availability of prescription drugs in Lebanon: A critical analysis of alternative policy options. Health Res. Policy Syst..

[32] von Elm E., Altman D.G., Egger M., Pocock S.J., Gøtzsche P.C., Vandenbroucke J.P., Initiative S. (2007). The Strengthening the Reporting of Observational Studies in Epidemiology (STROBE) Statement. Lancet.

[33] Ranganathan P., Deo V., Pramesh C.S. (2024). Sample size calculation in clinical research. Perspect. Clin. Res..

[34] Charan J., Biswas T. (2013). How to calculate sample size for different study designs in medical research?. Indian J. Psychol. Med..

[35] WHO (2019). Roadmap for Access to Medicines, Vaccines and Health Product 2019–2023. Comprehensive Support for Access to Medicines, Vaccines and other Health Products.

[36] Oldfield L., Penm J., Mirzaei A., Moles R. (2025). Prices, availability, and affordability of adult medicines in 54 low-income and middle-income countries: Evidence based on a secondary analysis. Lancet Glob. Health.

[37] WHO (2022). Health Systems Resilience Toolkit: A WHO Global Public Health Good to Support Building and Strengthening of Sustainable Health Systems Resilience in Countries with Various Contexts.

[38] Bhanja M., Vaishnaw A. (2025). Access to essential medicines in low-resource settings: A systematic review. J. Neonatal Surg..

[39] Lane J., Nakambale H., Kadakia A., Dambisya Y., Stergachis A., Odoch W.D. (2024). A systematic scoping review of medicine availability and affordability in Africa. BMC Health Serv. Res..

[40] Ozawa S., Shankar R., Leopold C., Orubu S. (2019). Access to medicines through health systems in low- and middle-income countries. Health Policy Plan..

[41] Witter S., Sheikh K., Schleiff M. (2022). Learning health systems in low-income and middle-income countries: Exploring evidence and expert insights. BMJ Glob. Health.

[42] Rowe A.K., Rowe S.Y., Peters D.H., Holloway K.A., Ross-Degnan D. (2021). The effectiveness of training strategies to improve healthcare provider practices in low-income and middle-income countries. BMJ Glob. Health.

[43] Petro E., Siebers A.C.M., Mantel-Teeuwisse A.K., Suleman F., van den Ham H.A. (2025). Availability and Affordability of Essential Medicines from the WHO Global Core List in Albania: A Cross-Sectional Survey Using the WHO/HAI Methodology. medRxiv.

[44] Menang O., Kuemmerle A., Maigetter K., Burri C. (2023). Strategies and interventions to strengthen pharmacovigilance systems in low-income and middle-income countries: A scoping review. BMJ Open.

[45] Das S., Khare S., Eriksen J., Diwan V., Stålsby Lundborg C., Skender K. (2024). Interventions on informal healthcare providers to improve the delivery of healthcare services in low-and middle-income countries: A systematic review. Front. Public Health.

[46] Menang O., van Eeuwijk P., Maigetter K., Stergachis A., Burri C. (2025). Pharmacovigilance processes in low- and middle-income countries: Moving from data collection to data analysis and interpretation. Ther. Adv. Drug Saf..

[47] Lamba G., Shroff Z.C., Babar Z.U., Ghaffar A. (2021). Drug shops for stronger health systems: Learning from initiatives in six LMICs. J. Pharm. Policy Pract..

[48] Lewandowski M., Religioni U., Świetlik D., Kobayashi A., Czech M., Wierzbiński P., Śliż D., Wierzba W., Plagens-Rotman K., Merks P. (2025). Perception of Generic Drugs Among Pharmacists in Poland: The Role of Sociodemographic Factors in Shaping Professional Attitudes and Practices. Healthcare.

[49] Mohd Sani N., Aziz Z., Panickar R., Kamarulzaman A. (2022). Pharmacists’ Perspectives of Biosimilars: A Systematic Review. BioDrugs Clin. Immunother. Biopharm. Gene Ther..

[50] Ntais C., Talias M.A., Fanourgiakis J., Kontodimopoulos N. (2024). Managing Pharmaceutical Costs in Health Systems: A Review of Affordability, Accessibility and Sustainability Strategies. J. Mark. Access Health Policy.

[51] World Health Organization, Organisation for Economic Co-operation and Development, World Bank Group (2018). Delivering Quality Health Services: A Global Imperative for Universal Health Coverage.

[52] Oldfield L., Penm J., Moles R. (2024). Exploring access to essential medicines in the South Pacific: Insights from a multi-country cross-sectional study. Lancet Reg. Health West. Pac..

