# Supplementary material for: Implementation Gaps in Public Outpatient Drug Programs: A Survey of Physicians in Urban Primary Care in Kazakhstan
Source: Int J Environ Res Public Health. 2026 Feb 24;23(3):279. doi: 10.3390/ijerph23030279 (PMC13026545; doi:10.3390/ijerph23030279)
Supplement: Supplementary file 1 [file ijerph-23-00279-s001.zip › ijerph-4128854-supplementary.pdf]

Supplementary materials

Supplementary Table S1. Mapping of study variables to CFIR domains

CFIR domain	CFIR construct (illustrative)	Study variable / indicator
Characteristics of individuals	Knowledge and beliefs about the intervention	Physician awareness of outpatient drug cost compensation mechanisms
Characteristics of individuals	Professional role and identity	Medical specialty (GPs, pediatricians, therapists, other specialists)
Characteristics of individuals	Individual experience	Length of professional experience
Inner setting	Communication networks	Sources of information on free medicines (physicians, posters, media)
Inner setting	Available resources	Availability of medicines and reported waiting time
Inner setting	Implementation climate	Frequency of prescription refusal due to system constraints
Implementation process	Executing	Physician responses to medicine shortages (recommend purchase, wait, substitute)
Implementation process	Reflecting and evaluating	Patient complaints and feedback pathways as reported by physicians

Supplementary Table S2. STROBE Checklist for Cross-Sectional Studies

Section	Item No.	Recommendation	Addressed in manuscript
**Title and Abstract**	1a	Indicate the study's design in the title or abstract	Title; Abstract (Methods)
	1b	Provide an informative and balanced summary of what was done and what was found	Abstract
**Introduction**	2	Explain the scientific background and rationale for the investigation	Introduction
	3	State specific objectives, including any prespecified hypotheses	End of Introduction

**Methods**	4	Present key elements of study design early in the paper	Section 2.2 Study design
	5	Describe the setting, locations, and relevant dates	Section 2.2 Study setting
	6a	Give eligibility criteria and sources and methods of selection of participants	Section 2.3 Study population
	7	Clearly define all outcomes, exposures, predictors, and confounders	Section 2.8 Outcome and explanatory variables
	8	Give sources of data and details of methods of assessment	Sections 2.6 and 2.7
	9	Describe efforts to address potential sources of bias	Sections 2.6, 2.7
	10	Explain how the study size was arrived at	Section 2.4 Sample size determination
	11	Explain how quantitative variables were handled in analyses	Section 2.9 Statistical analysis
	12a	Describe all statistical methods, including those used to control for confounding	Section 2.9
	12b	Describe any methods used to examine subgroups and interactions	Section 2.9
	12c	Explain how missing data were addressed	Section 2.7
	12d	Describe analytical methods taking account of sampling strategy	Section 2.9
	12e	Describe any sensitivity analyses	Not applicable (descriptive study)
**Results**	13a	Report numbers of individuals at each stage of study	Section 3.1
	13b	Give reasons for non-participation at each stage	Section 2.7
	13c	Consider use of a flow diagram	Not applicable
	14a	Give characteristics of study participants	Table 1; Section 3.1
	14b	Indicate number of participants with missing data for each variable	Section 2.7

	15	Report numbers of outcome events or summary measures	Sections 3.2–3.6
	16	Give unadjusted estimates and precision (e.g., 95% CI)	Section 3.6
	17	Report other analyses performed	Section 3.6
**Discussion**	18	Summarize key results with reference to study objectives	Section 4 Discussion
	19	Discuss limitations of the study	Limitations
	20	Give a cautious overall interpretation of results	Discussion
	21	Discuss the generalizability of the study results	Discussion
**Other information**	22	Give source of funding and role of funders	Funding statement

Supplementary Table S3. Physician questionnaire on outpatient drug provision (English version)

Section	Item No.	Survey question (English)	Response options
**I. General characteristics**	1	Sex	Male / Female
	2	Age group (years)	<30; 30–39; 40–49; 50–59; ≥60
	3	Medical specialty	General practitioner; Therapist; Pediatrician; Other (specify)
	4	Length of professional experience (years)	Open-ended
**II. Awareness and prescribing practices related to the ALO program**	5	In your opinion, where do patients obtain information about their entitlement to free or subsidized medicines under the ALO program?	I do not know; From the treating physician; From other physicians; From information boards in the clinic; From relatives/friends; Mass media / Internet
	6	How often do you have to refuse issuing a prescription under the ALO program?	Always; Often; Rarely; Never; Difficult to answer
	7	If you refuse to issue a prescription under the ALO program, what are the main reasons? (multiple answers possible)	Medicine unavailable in pharmacy; Not included in the free provision list; Recommend purchase at patient's expense; Pending specialist consultation;

			Pending diagnostic examination; Other (specify)
	8	How long do patients usually wait to receive medicines under the ALO program?	Immediately; Up to 2 weeks; Up to 1 month; Up to 3 months; More than 3 months
	9	How often do patients interrupt or discontinue treatment due to unavailability of medicines under the ALO program?	Always; Almost always; Sometimes; Rarely; Never
	10	What actions do you usually take when free medicines are unavailable?	Recommend purchasing independently; Prescribe an alternative medicine; Advise waiting until availability; Other (specify)
	11	Do patients submit complaints about the lack of free medicines, and where are these complaints most often addressed?	Head physician; Patient support service; City/regional health authority; Ministry of Health; Presidential website
**III. Patient behavior and policy perceptions**	12	How often do patients insist on continuing therapy with the original (brand-name) medicine?	Always; Often; Sometimes; Never; Difficult to answer
	13	How do patients generally respond to the prescription of generic medicines?	Never refuse; Rarely refuse; Sometimes; Often; Always refuse; Difficult to answer
	14	Would you support informing patients about medicine availability under the ALO program via SMS, WhatsApp, or phone calls?	Yes; No; Difficult to answer
	15	Would you support a reimbursement model in which patients purchase medicines in any pharmacy and receive 50-70% cost compensation from the health insurance fund or clinic?	Yes; No; Difficult to answer

Note: This questionnaire was developed specifically for the present study, administered in Russian and Kazakh. The table presents the harmonized English version for international publication.
